# datasets on employee value proposition (evp) and performance of selected fast moving consumer goods (FMCGs) firms in Nigeria

**DOI:** 10.1016/j.dib.2018.06.027

**Published:** 2018-06-27

**Authors:** Odunayo Salau, Adewale Osibanjo, Anthonia Adeniji, Oluwatunmise Ojebola, Olumuyiwa Oludayo, Hezekiah Falola, Tolulope Atolagbe

**Affiliations:** Covenant University, Nigeria

**Keywords:** EVP, Retention, Reward, Satisfaction, Performance

## Abstract

The article presented an integrated dataset on employee value proposition (EVP) and performance of selected Fast Moving Consumer Goods (FMCGs) firms in Nigeria. The study adopted quantitative approach with a descriptive research design to establish the major determinants of employee value proposition. The population of this study included staff and management of the selected firms. Data was analysed with the use of measurement and structural equation modelling and the field data set is made widely accessible to enable critical or a more comprehensive investigation. The findings identified career growth and reward flexibility as predictive determinants of EVP for increased performance of sampled firms. It was recommended that FCMGs firms need to adopt consistent range of strategies to improve company strength and ethical culture for performance to be heightened.

## 1 Introduction

In today׳s dynamic and competitive work environment, organizations are faced with challenges of how to attract and retain productive workforce. This however, has made organizations to look beyond remuneration but also consider factors like work culture, career growth, work life balance, training and development and others to create proposition that is valued by employees. As a result, organizations now use employee value proposition (EVP) as a strategic tool to attract, develop, retain best talents and achieve competitive advantage.

Employee Value Proposition (EVP), in this context, is the unique set of benefits which an employee receives in return for the skills, capabilities and experience they bring to a company. However, many industry analysts attribute the incessant attrition rate and poor performance of firms to lack of awareness of the application of motivational strategies. EVP has become very relevant, especially in the new millennium and have received overwhelming dominance from the developed countries and of late a focus on the transitional or emerging economies. Hence, this paper therefore explores in detail the dimensions and implications of EVP on the performance of firms in developing countries, such as, Nigeria and from a tripartite perspective.

**Specification Table**

**Table:** 

**Subject area**	Strategic HRM
**More Specific Subject Area**	Talent Management
**Type of Data**	Primary data
**How Data was Acquired**	Mainly through questionnaire
**Data format**	Raw, analyzed, Inferential statistical data
**Experimental Factors**	Population comprises sampled Fast Moving Consumer Goods firms in Nigeria. The researcher-made questionnaire which contained data on employee value proposition (EVP) and job performance
**Experimental features**	Creating and implementing motivational strategies that are valued by employees is important and is an essential component for success in an increasingly competitive environment.
**Data Source Location**	Fast Moving Consumer Goods (FMCGS) Firms, Lagos, Nigeria
**Data Accessibility**	Data is included in this article

**Value of Data**

• The data can be used by managers to properly make decisions that in the long-run would lead to goal attainment in the organization.
• The data can be used to enlighten managers on the importance of employee value proposition and how it can be beneficial to overall performance of the organization.
• The data provides ample knowledge on how different employee value proposition attributes would in turn lead to organizational success
• The data described in this article is made widely accessible to facilitate critical or extended analysis.

## 2 Data

**Fig. 1:**
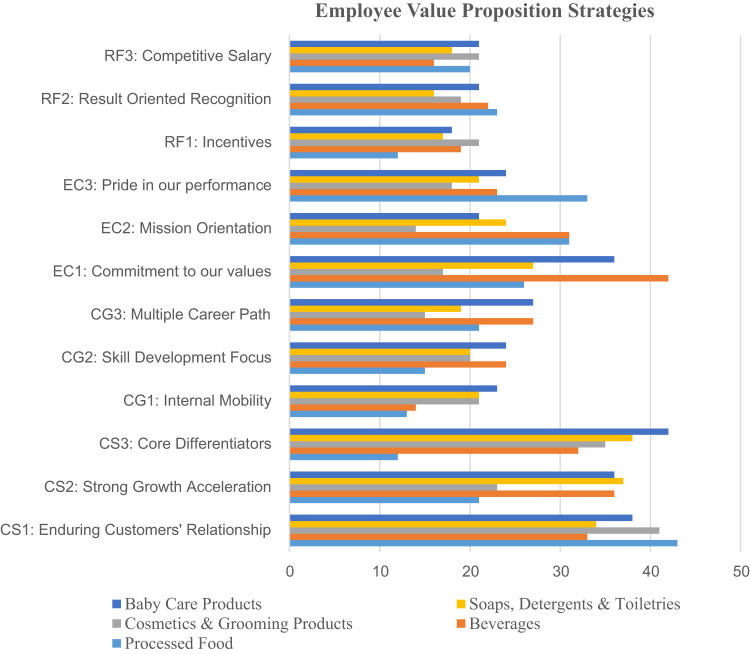
A detailed description of Employee Value Proposition Strategies.

The study employed quantitative (survey) method to collect data from staff and management of sampled firms. The decision to elicit information from the employees and the management group was based on the fact that while employees were often in the best position to describe their real employment relationships and knowledge of retention practices; it is also crucial to investigate these practices from the perceptions of the managers. This shows that the samples were diverse and it can be concluded that non-response bias will not significantly affect the generalizability of the study findings. The scales are derived from previous studies. They are validate and modified to fit the current research context. Due to the limitation of space, details of questionnaire are omitted. Following recommended two-stage analytical procedures [2], confirmatory factor analysis and structural model were tested sequentially. Specifically, the model fits were assessed and the results of measurement model test were reported in Table 1 and a detailed description was EVP strategies was provided in Fig. 1.

**Table 1:** demonstrated measurement model using Confirmatory Factor Analysis (CFA) to assess the composite reliability and the average variance extracted (AVE).

**Measurement**	**Loading**	**Indicator reliability**	**Error variance**	**Compose reliability**	**Ave. variance estimated**
**Employee Value Proposition**	**> 0.7**		**< 0.5**	**> 0.8**	**> 0.5**
a. **Company Strength**					
CS1: Enduring Customers׳ Relationship	0.823	0.6773	0.3227	0.8730	0.6962
CS2: Strong Growth Acceleration	0.835	0.6972	0.3028		
CS3: Core Differentiators	0.845	0.7140	0.2860		
b. **Career Growth**					
CG1: Internal Mobility	0.828	0.6856	0.3144	0.8731	0.6966
CG2: Skill Development Focus	0.864	0.7465	0.2535		
CG3: Multiple Career Path	0.811	0.6577	0.3423		
c. **Ethical culture**					
EC1: Commitment to our values	0.876	0.7674	0.2326	0.8786	0.7074
EC2: Mission Orientation	0.793	0.6288	0.3712		
EC3: Pride in our performance	0.852	0.7259	0.2741		
d. **Reward flexibility**					
RF1: Incentives	0.805	0.6480	0.3520	0.8876	0.6639
RF2: Result Oriented Recognition	0.828	0.6856	0.3144		
RF3: Competitive Salary	0.815	0.6642	0.3358		
RF4: Flexible work environment	0.811	0.6577	0.3423		

Note: The results above have proven that the data are good in terms of the CFA.

**Fig. 2:**
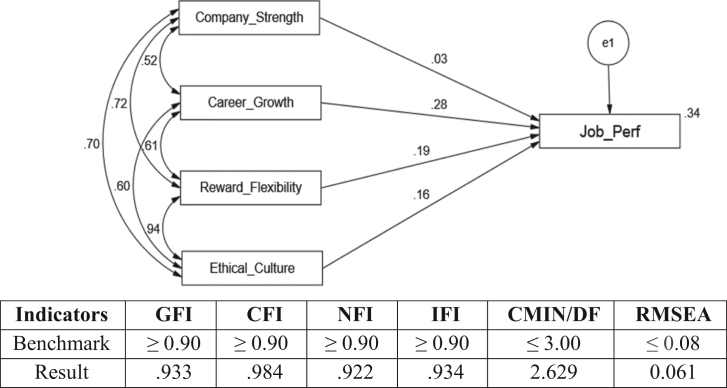
Standardized regression weights.

The model fit was tested to show the regression weight and several fit indices between observed and unobserved as presented in Fig. 2.

The result indicates that there are varying explanations for the dependent variables. Hence, it is seen that the strength of regression weights of paths has a strong direction.

Basically, this study revealed that employee value proposition (EVP) has significant and positive impact on job performance. The study revealed that sampled firms offer a compelling employee value proposition anchored by four key elements: company strength, career growth, an ethical culture, and rewards & flexibility. The findings identified career growth and reward flexibility as predictive determinants of EVP for increased performance of sampled firms. It was recommended that FCMGs firms need to adopt consistent range of strategies to improve company strength and ethical culture for performance to be heightened. The success of any organisation depends on the degree of multiple options for keeping skills current and planning future moves with a wide range of benefits to promote health and wellness, building financial security and allowing for personal choice hence, this present study has extensive implications for both the managers, employees, government, educators and researchers in this regard. To this end, the data presented in this article is imperative for more comprehensive analysis or investigation.

## 3 Experimental design, materials and methods

Of 402 copies of questionnaire were distributed, only 361 responses were received resulting in a response rate of 89.8%. Staff of selected Fast Moving Consumer Goods firms were represented in this study. Data were gathered from directors, managers, assistant managers, scientists, field agents, and other categories of employees across the sampled firms with the aid of a researcher- made questionnaire based on the works of [1], [3], [4], [5], and [6]. The demographic data presented information based on gender, age, education and experience as well as questions related to employee value proposition and performance. The collected data were coded and analysed using SPSS version 22. Data was analysed through the measurement model and structural model. Importantly, the participants were selected based on the following inclusion criteria:

### 3.1 Inclusion criteria

• Participants had to be staff of the sampled FCMGs firms
• Participants must be literate, able to read and write English
• Participants must have signed the consent form provided
• Participants must have worked with the firm for a minimum period of 3 years

As regards EVP, items used included: the main reasons for participants enduring customers׳ relationship; whether a detailed job description was given on appointment with the organization, and if the job description gives strong growth acceleration; the degree of internal mobility and skill development focus; relevance of multiple career path and mission orientation; extent of commitment to values and pride in performance; and the direction of reward flexibility. The section on job performance was adapted from a previously validated questionnaire – the Employee Performance Questionnaire, EPQ. The researchers ensured that respondents were well informed about the background and the purpose of this research and they were kept abreast with the participation process. Respondents were offered the opportunity to stay anonymous and their responses were treated confidentially.
