# Supplementary material for: datasets on employee value proposition (evp) and performance of selected fast moving consumer goods (FMCGs) firms in Nigeria
Source: Data Brief. 2018 Jun 27;19:1907–11. doi: 10.1016/j.dib.2018.06.027 (PMC6141159; doi:10.1016/j.dib.2018.06.027)
Supplement: Supplementary file 1 — Supplementary material [file mmc1.docx]

**DECLARATION OF INTEREST FORM**

**DATASETS** [**ON EMPLOYEE VALUE PROPOSITION (EVP) AND PERFORMANCE OF SELECTED FAST MOVING CONSUMER GOODS (FMCGs) FIRMS IN NIGERIA**](https://www.sciencedirect.com/science/article/pii/S2352340917302949)

Odunayo **SALAU**; Covenant University

Adewale **OSIBANJO**; Covenant University

Anthonia **ADENIJI**; Covenant University

Oluwatunmise **OJEBOLA**; Covenant University

Olumuyiwa **OLUDAYO**; Covenant University

Hezekiah **FALOLA**; Covenant University

Tolulope **ATOLAGBE**; Covenant University

We, the Authors of paper entitled above certify that we have seen and approved the final version of the manuscript being submitted. This is an original work and has not received prior publication and is not under consideration for publication elsewhere. It is also important to state that there is no financial/personal interest or belief that could affect our objectivity and to prevent ambiguity, we humbly want to state explicitly that there is no conflicts of interest as regards the review and publication of this paper.

Thank you.

SALAU Odunayo Paul

*Signed*
